# The expression of LC-3 is related to tumor suppression through angiogenesis in esophageal cancer

**DOI:** 10.1007/s12032-013-0701-x

**Published:** 2013-10-13

**Authors:** Toshihide Sakurai, Hiroshi Okumura, Masataka Matsumoto, Yasuto Uchikado, Tetsuro Setoyama, Itaru Omoto, Tetsuhiro Owaki, Kosei Maemura, Sumiya Ishigami, Shoji Natsugoe

**Affiliations:** Department of Digestive Surgery, and Breast and Thyroid Surgery, Kagoshima University Graduate School of Medical and Dental Sciences, Sakuragaoka 8-35-1, Kagoshima, 890-8520 Japan

**Keywords:** LC3, Autophagy, Esophageal cancer, Angiogenesis

## Abstract

Autophagy is important in the development and remodeling of cells. It is required for cellular adaptation to nutrient deprivation and elimination of damaged organelles. Recently, autophagy has been implicated in carcinogenesis and metastasis. We hypothesized that autophagy-related proteins are initiated until nutrition is supplied by angiogenesis. We evaluated the clinicopathological significance of LC3, an autophagic marker, and its relationship to angiogenesis in patients with esophageal squamous cell carcinoma (ESCC). We immunohistochemically investigated the expression of LC3 as well as endoglin (CD105), a microvessel marker, and vascular endothelial growth factor A (VEGF-A) in 142 patients with ESCC. The high, moderate, and low expression rates of LC3 were 40, 31, and 29 %. LC3 expression inversely correlated with depth of invasion, lymph node metastasis, lymphatic invasion, MVD, VEGF-A expression, and poor prognosis. The overall survival rate was better in patients with high LC3 expression compared to patients with low LC3 expression. We demonstrate that low LC3 expression is related to tumor development as facilitated by angiogenesis and that alteration in LC3 expression is closely related to prognosis. Expression of LC3 proteins is a useful marker for determining tumor prognostic behavior in patients with ESCC.

## Introduction

Programmed cell death (PCD) is a crucial mechanism regulating cell death and homeostasis and involves two processes, apoptosis and autophagy. Apoptosis, or type I PCD, is a caspase-dependent process. Autophagy, or type II PCD, leads to bulk degradation of intracellular components induced by cellular starvation and other metabolic stresses [1, 2].

Autophagy is important in the development and differential remodeling of cells, and is required for the cellular adaptation to nutrient deprivation and elimination of damaged organelles [3]. Furthermore, autophagy has a role in the elimination of pathogens [4] and the contribution to dead-cell clearance during apoptosis [5]. It is known that when a cell begins to starve, autophagy-related proteins are initiated. In yeast, more than 30 autophagy-related genes (ATG) encoding the protein executing autophagy have been identified [6]. Among these proteins, microtubule-associated process 1 light chain 3 (LC3), the mammalian homologue of yeast 8, is a key regulator involved in forming autophagosomes [7].

LC3 exists in two forms, LC3-I and LC3-II (a LC3-phospholipid conjugate). LC3-I is localized in the cytoplasm under non-stress stimulation. In the first step of autophagy, an isolation membrane is formed consisting of two parallel lipid layers. The isolation membrane then encircles the cytoplasmic components and the edges of each membrane fuse. This vesicular structure is called autophagosome. The autophagosome is a double-membrane structure that non-selectively surrounds the cytoplasmic contents and fuses with the lysosome membrane. The contents of the autophagosome are then degraded by the lysosomal enzymes. The stress of starvation immediately converts LC3-I to LC3-II and LC3-II then binds to the autophagosome. Therefore, endogenous LC3 expression is felt to be a marker of autophagy [8–10].

Recent research suggests that autophagy is involved in carcinogenesis and metastasis of cancer, although there are few reports regarding LC3 expression in cancers involving the digestive tract.

We hypothesize that autophagy-related proteins are initiated when cancer cells begin to starve until nutrition is supplied by angiogenesis. In the present study, in order to understand the relationship between autophagy and angiogenesis, we examine microvessel density (MVD) using endoglin (CD105) expression as a microvessel marker. We also study angiogenesis using vascular endothelial growth factor (VEGF-A) expression. Since the expression of LC3 is strongly associated with venous invasion, we immunostain for CD105 to determine MVD since CD105 is useful marker for the development of new blood vessels induced by tumors [11–14] and angiogenesis is related to VEGF-A expression [15, 16].

The aims of this retrospective study were to evaluate the clinicopathological significance of LC3 expression and to examine the relationship between autophagy and angiogenesis in patients with esophageal squamous cell carcinoma (ESCC).

## Materials and methods

### Study groups

Our study was approved by the institutional review board of our university. All patients included in our study gave their informed written consent. One hundred and forty-two consecutive patients (130 males and 12 females) with ESCC who underwent curative surgery at Kagoshima University Hospital between 1996 and 2003 were included in this retrospective study. All patients underwent an esophagectomy with lymph node dissection. No patient in our study had endoscopic mucosal resection, palliative resection, preoperative chemotherapy, or radiotherapy, and no one had synchronous or metachronous cancer in other organs. The age of our patients ranged from 38 to 86 years (mean 64.7 years).

Clinicopathological classification of our patients’ tumors was based on the tumor-node-metastasis classification for esophageal carcinoma from the international union against cancer [17]. Histologically, 41 patients had well-differentiated, 76 had moderately differentiated, and 25 had poorly differentiated squamous cell carcinoma. Twenty-six tumors were located in the upper third, 72 in the middle third, and 44 in the lower third of the esophagus. According to the depth of tumor invasion, 57 patients had pathological (p) T1 (40.1 %), 22 had pT2 (15.5 %), 54 had pT3 (38.0 %), and 9 had pT4 (6.4 %). Lymph node metastases (pN1) were found in 81 (57.0 %) of the 142 patients. Distant metastases (pM1) were found in 27 (19.0 %) of the 142 patients. All of the M1 tumors were due to distant lymph node metastases. Lymphatic invasion was found in 69.7 % (99/142), and venous invasion was found in 57.0 % (81/142). All patients were followed up after discharge with a radiographic examination every 1–3 months, computed tomography every 3–6 months, and ultrasonography every 6 months. Follow-up data after treatment were collected from all patients with a median follow-up period of 41 months (range 1–137 months). The clinicopathologic features of the study group are summarized in Table 1.

**Table 1:** Relationship between LC3 expression and clinicopathologic variables

	Total *n* = 142	LC3 expression			*P* value
		High *n* = 57	Moderate *n* = 44	Low *n* = 41	
Age		64.5 ± 7.6	67.2 ± 9.2	63.1 ± 9.6	0.42
Gender					
Male	130	51	42	37	0.76
Female	12	6	2	4	
Turn or location					
Upper third	26	14	7	5	0.77
Middle third	72	26	23	23	
Lower third	44	17	14	13	
Histology					
Well	41	18	10	13	0.51
Moderate	76	33	25	18	
Poor	25	6	9	10	
pT					
pTl	57	36	16	5	<0.0001
pT2	22	9	7	6	
pT3	54	9	18	27	
pT4	9	3	3	3	
pN					
pN0	61	28	22	11	0.04
pNl	81	29	22	30	
pM					
pM0	115	51	34	30	0.09
pMl	27	6	10	11	
Stage					
I	37	22	12	3	0.0001
II	43	20	13	10	
III	35	9	9	17	
IV	27	6	10	11	
Lymphatic invasion					
Negative	43	22	16	5	0.02
Positive	99	35	28	36	
Venous invasion					
Negative	61	36	17	8	0.0001
Positive	81	21	27	33	

### Immunohistochemistry

Tumor samples were fixed with 10 % formaldehyde in phosphate-buffered saline (PBS), embedded in paraffin, and sectioned into 4-μm-thick slices. They were deparaffinized in xylene and dehydrated in graded ethanol. For antigen retrieval, sections were autoclaved in 10 mM citrate buffer solution for 10 min at 120 °C and allowed to cool at room temperature. The endogenous peroxidase activity of specimens was blocked by immersing the slides in a 3 % H2O2 solution for 30 min. After washing three times with PBS for 5 min each, the sections were treated with 3 % bovine serum albumin for 30 min at room temperature. The blocked sections were incubated overnight at 4 °C with rabbit anti-LC3 monoclonal antibody (ABGENT) diluted 1:200, and were incubated for 1 h at room temperature with anti-CD105 mouse monoclonal antibody (DAKO Corporation, Carpinteria, CA, USA) diluted 1:100, and purified rabbit polyclonal antihuman VEGF (A-20 Santa Cruz Biotechnology, Inc, CA, USA) diluted 1:200 followed by staining with a streptavidin–biotin peroxidase kit (Nichirei, Tokyo, Japan). The sections were thrice washed in PBS for 5 min and the immune complex was visualized by incubating the sections with diaminobenzidine tetrahydrochloride. Then, sections were counterstained with hematoxylin. The nerve tissues were used as a positive control for LC3 [18]. The negative controls were performed by replacing the primary antibodies with PBS.

### Evaluation of LC3 expression

Evaluation of immunohistochemistry was independently performed by two investigators (T.S. and H.O.) who were blind to all patients’ information. The estimation of LC3 immunoreactivity was performed according to the intensity and percentage of positive stained cell as previously reported [18]. The intensity of immunoreactivity was graded into three phases, they are 0: negative, 1: weak, and 2: strong. The percentage of stained cells was graded as 0: 0–50 % and 1: 51–100 %. We designated “High” expression as tumors that stained strongly over 51–100 % of the expression area and as “Low” those tumors that stained negative. The other tumors were designated as “Moderate” tumor expression.

### Evaluation of MVD

Vessel count was assessed using light microscopy in those areas of the tumor containing the highest numbers of capillaries and small venules at the invasive edge. These highly vascular areas were identified by scanning tumor sections at low power (× 40 and × 100). After six areas were identified, vessel count was performed in a × 200 field and the average of six areas was determined as MVD. As described by Weidner et al. [19], identification of a vessel lumen was not necessary for a structure to be defined as a vessel.

### Evaluation of VEGF-A

Five fields were viewed at low power (× 40 and × 100), and predominant VEGF intensity was scored in the range from 0 to 3. We defined score of 3 + as a strongest stain (positive control) and score of 0 as no detectable stain (negative control) as reported previously [20]. In our study, we designated as “Positive” expressed tumors those which stained 3 + . The other tumors were designated as “Negative” tumors.

### Statistical analysis

Dates were analyzed using the *χ* ^2^ test or Student’s *t* test for statistical significance. The Kaplan–Meier method was used for survival analysis, and the differences were estimated using the log rank test. Prognostic factors were examined by univariable and multivariable analyses. The *p* value was considered significant if < 0.05. All statistical analyses were performed using stat view 5.0 (Abacus Concepts, Berkeley, CA, USA).

## Results

### Expression of LC3 in esophageal squamous cell carcinoma

**Fig. 1:**
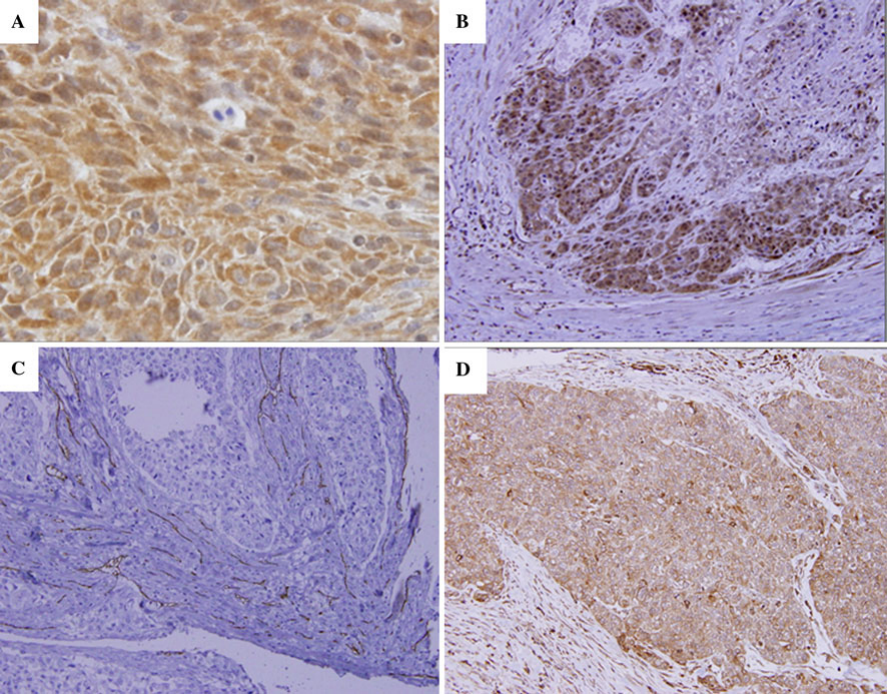
Expression of LC3, CD105, and VEGF-A protein in esophageal squamous cell carcinoma. **a** Positive expression of LC3 in the cytoplasm of ESCC (× 200). **b** Expression of LC3 is stronger at the invasive front of a tumor (× 100). **c** Intratumoral blood microvessels detected as CD105 positive in single or clustered endothelial cells with or without lumen (× 100). **d** The positive expression of VEGF-A is seen in the cytoplasmic regions (× 100)

High expression of LC3 was found in the cytoplasm of 40.1 % of ESCC cells (57/142), moderate expressions was found in 31.0 % (44/142), and low expression was found in 28.9 % (41/142) of ESCC cells. The invasive front of the tumor had stronger LC3 expression compared to other areas of the tumor, especially in the T1 stage (Fig. 1a, b).

### Relationship between the expression of LC3 and clinicopathological findings

LC3 expression was inversely related to the following clinicopathologic parameters: depth of tumor invasion, stage, lymph node metastasis, lymphatic invasion, and venous invasion (Table 1). Compared to tumors with high and moderate LC3 expressions, tumors with low LC3 expression showed deeper invasion, were of a more advanced stage, showed positive lymph node metastasis, and displayed both lymphatic and venous invasion (*p* < 0.0001, *p* = 0.0001, *p* = 0.04, *p* = 0.02 and *p* = 0.0001, respectively).

### Expression of LC3 and MVD in esophageal squamous cell carcinoma

**Fig. 2:**
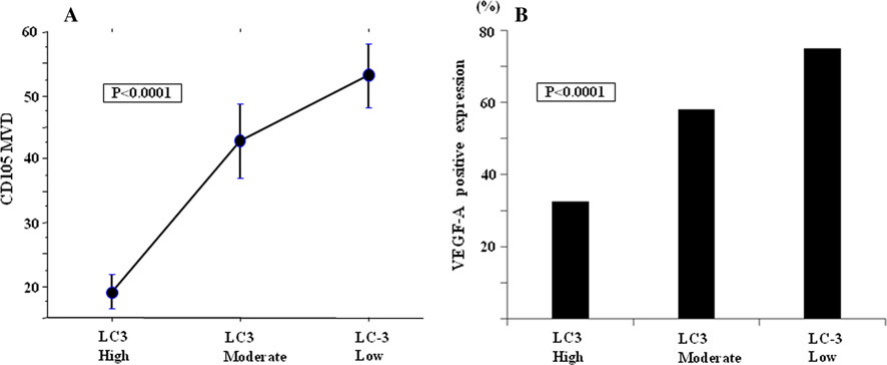
Expression of LC3 and MVD in esophageal squamous cell carcinoma. **a** The LC3 expression becomes significantly weaker with increases in MVD (*p* < 0.0001). The median MVD is 19.1 ± 10.3 in high LC3 expression tumor, 40.9 ± 20.1 in moderate expression tumor, and 54.0 ± 15.2 in low expression tumor. **b** The LC3 expression becomes significantly weaker with increases in ratio of VEGF-positive expression (*p* < 0.0001). Whereas 32 % of high LC3 expression tumor revealed positive expression of VEGF-A, 78 % of low LC3 expression tumor revealed positive expression of VEGF-A

CD105 was detected in blood endothelial cells as shown in Fig. 1c. The median MVD was 36.7 ± 21.0 among all tumors, and the tumors with LC3 high expression had low MVD (*p* < 0.0001). The median MVD was 19.1 ± 10.3 in tumors with high LC3 expression, 40.9 ± 20.1 in tumors with moderate expression, and 54.0 ± 15.2 in low LC3 expression tumor. A significant inverse relationship was found between LC3 expression and MVD (*p* < 0.0001) (Fig. 2a).

### Expression of LC3 and VEGF-A in esophageal squamous cell carcinoma

The expression of VEGF-A was detected in the cytoplasm of ESCC cells (Fig. 1d). The expression of LC3 was significantly associated with VEGF-A expression. Tumors with low LC3 expression had stronger VEGF-A expression than tumors with high and moderate LC3 expressions (*p* < 0.0001) (Fig. 2b). Whereas the positive expression of VEGF-A was showed in 32 % among the LC3 high expression tumors, it was showed 78 % among the LC3 low expression tumors.

### Relationship between expression of LC3 and prognosis

**Fig. 3:**
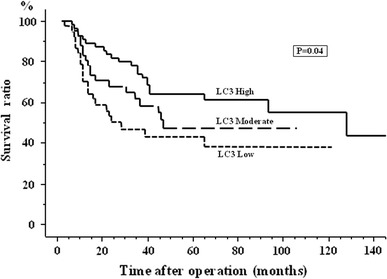
Postoperative overall survival curves according to the expression of LC3. The patients with higher LC3 expression tumors have longer overall survival than with lower LC3 expression tumors (*p* = 0.04)

The expression of LC3 was significantly associated with overall survival (Fig. 3). Patients with high LC3 expression had longer overall survival than those with low LC3 expression (*p* = 0.04).

### Univariate and multivariate analyses of survival

Univariate analysis showed that the following factors had a significant impact on postoperative survival: low LC3 expression, depth of tumor invasion, the presence of lymph node metastasis, lymphatic invasion, and venous invasion. Multivariate regression analysis indicated that depth of tumor invasion was the only independent prognostic factor (Table 2).

**Table 2:** Univariate and multivariate analyses of prognostic factors in esophageal squamous cell carcinoma

Prognostic factor	Univariate *P*	Multivariate *P*	Hazard ratio (95 % confidence interval)
LC3 (high/moderate/low)	0.030	0.607	0.874 (0.523–1)
pT (1/2/3/4)	<0.0001	0.0001	0.163 (0.068–0.389)
pN (pN0/l)	0.001	0.352	1.394 (0.692–2.807)
Venous invasion (negative/positive)	<0.0001	0.190	0.685 (0.389–1.207)
Lymphatic invasion (negative/positive)	0.002	0.341	0.679 (0.305–1.508)

## Discussion

We correlated LC3 expression and clinicopathological factors and prognosis in 142 patients with ESCC. We found that high or moderate expression of LC3 was observed in 71 % of ESCC tumors. This result is similar to the previous immunohistochemical studies on gastrointestinal carcinoma in which LC3 expression was detected in the majority of esophageal, gastric, and colorectal cancers [21]. Other reports have found even higher LC3 positive expression in colorectal cancers with 90 % of tumors demonstrating LC3 expression regardless of the strength of expression [10]. In addition, in pancreatic cancer, the positive expression of LC3 was 87.3 % in the periphery and 76.0 % in the central area of tumor [18]. These differences in frequency of expression may be due, in part, to different evaluation criteria used or differences in overall tumor area demonstrating positive LC3 expression. But, even if there were differences in evaluation methods, these high frequencies suggest that autophagy is closely associated with tumor.

In the present study, low LC3 expression showed a strong correlation with depth of tumor invasion, stage, venous invasion, MVD, and VEGF-A expression. The expression of LC3 in T1 stage carcinoma at the invasive front was higher compared to more advanced stages of tumor growth. Yoshioka et al. [21] also reported a close relationship between LC3-positive expression, intraepithelial neoplasia, and T1 carcinoma in esophageal cancer. Furthermore, we found that LC3 expression becomes less pronounced as cancer develops and tumors with high LC3 expression show less frequent VEGF expression and MVD. Thus, autophagy, as measured by LC3 expression, is inversely related to MVD and VEGF-A expression and this appears to be a novel finding.

**Fig. 4:**
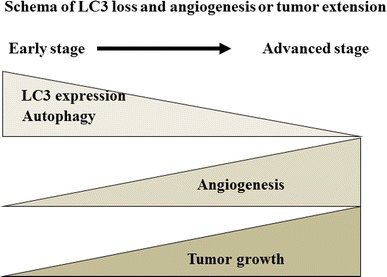
LC3, angiogenesis, and tumor growth in ESCC. The higher LC3 expression induces autophagy and suppresses angiogenesis and tumor invasion in the early phase of ESCC; in contrast, lower LC3 expression reduces autophagy and increases angiogenesis and tumor invasion in the advanced stage of ESCC

Autophagy is a self-recycling process involving the degradation of cytoplasmic organelles and proteins, which is induced by nutrient deprivation, growth factor deprivation, and hypoxia in normal cells [22]. In cancer cells, most of the nutrients and oxygen is needed for their own abnormal growth [23]. Therefore, tumors release vascular proliferation factors, such as VEGF, to promote angiogenesis [24, 25]. The expression of VEGF is associated with cancer development including esophageal carcinoma [26]. Since proliferation of tumor cells develops faster than the formation of new blood vessels, tumor cells are rapidly exposed to an avascular environment [27]. This environment is similar to the autophagy induction system environment. Tumor cells with enhanced LC3 expression synchronously express carbonic anhydrase IX (CA IX) as a hypoxia marker [18]. In the phase of immature angiogenesis, autophagy is necessary for tumor development. These findings suggest that tumors with LC3-positive expression induce autophagy and suppress both angiogenesis and tumor invasion in the early phase of ESCC (Fig. 4).

Previous reports have correlated autophagy and digestive cancers investigating beclin-1 expression, an up-regulator of autophagy. Chang et al. reported that beclin-1 expression occurred at a relatively early stage of colorectal and gastric cancer [28]. They concluded that beclin-1 was a pro-tumor factor in cancer development. On the other hand, beclin-1-deficient mice suffer from a high incidence of spontaneous tumors such as lymphoma, lung adenocarcinoma, and hepatocellular carcinoma [29, 30]. Ding et al. [31] suggested that, in hepatocellular carcinoma, autophagic gene activity was suppressed. They concluded that beclin-1 was a tumor suppressor. Shen et al. [32] clarified that the positive rates of LC3 and beclin-1 were significantly higher in benign and borderline ovarian tumors compared to epithelial ovarian carcinoma. These reports suggest that the expression of ATG is downregulated in cancer and that the decrease in autophagic capacity relates to tumorigenesis and tumor development.

In our study, low LC3 expression, depth of tumor invasion, lymph node metastasis, lymphatic invasion, and venous invasion were all prognostic factors. Multivariate analysis revealed that the depth of tumor invasion was an independent prognostic factor. These findings confirm results from a recent study of pancreatic cancer, where a significant correlation was found between low LC3 expression and poor clinical outcome with shorter disease-free survival time [18]. In gastrointestinal cancer, postoperative survival was higher in the LC3-positive group compared to the LC3-negative group [21]. Regarding the role of autophagy in cancer development, LC3 is expressed in the early phase of ESCC. Therefore, patients with LC3-positive expression have a better prognosis. Further studies are necessary to clarify the mechanisms underlying these results.

In conclusion, we demonstrate that low LC3 expression is related to tumor development and that alteration of LC3 expression is closely related to prognosis. Autophagy may have a role in the suppression of tumor invasion by controlling angiogenesis at the invasion front of the tumor in the early phase of ESCC. LC3 expression appears to be a useful marker for determining malignant potential and clinical outcome in patients with ESCC.
